# The effect of wool hydrolysates on squamous cell carcinoma cells *in vitro*. Possible implications for cancer treatment

**DOI:** 10.1371/journal.pone.0184034

**Published:** 2017-08-31

**Authors:** Tatsiana Damps, Anna Katarzyna Laskowska, Tomasz Kowalkowski, Monika Prokopowicz, Anna Katarzyna Puszko, Piotr Sosnowski, Joanna Czuwara, Marek Konop, Krzysztof Różycki, Joanna Karolina Borkowska, Aleksandra Misicka, Lidia Rudnicka

**Affiliations:** 1 Department of Neuropeptides, Mossakowski Medical Research Centre, Polish Academy of Sciences, Warsaw, Poland; 2 Chair of Environmental Chemistry and Bioanalytics, Faculty of Chemistry, Nicolaus Copernicus University, Toruń, Poland; 3 Interdisciplinary Centre of Modern Technology, Nicolaus Copernicus University, Toruń, Poland; 4 Laboratory of Chemical Synthesis, CePT, Mossakowski Medical Research Centre, Polish Academy of Sciences, Warsaw, Poland; 5 Faculty of Chemistry, University of Warsaw, Warsaw, Poland; 6 Department of Dermatology, Medical University of Warsaw, Warsaw, Poland; 7 Department of Human Epigenetics, Mossakowski Medical Research Centre, Polish Academy of Sciences, Warsaw, Poland; University of Alabama at Birmingham, UNITED STATES

## Abstract

Squamous cell carcinoma of the skin is the second most common cutaneous malignancy. Despite various available treatment methods and advances in noninvasive diagnostic techniques, the incidence of metastatic cutaneous squamous cell carcinoma is rising. Deficiency in effective preventive or treatment methods of transformed keratinocytes leads to necessity of searching for new anticancer agents. The present study aims to evaluate the possibility of using wool hydrolysates as such agents. Commercially available compounds such as 5-fluorouracil, ingenol mebutate, diclofenac sodium salt were also used in this study. The process of wool degradation was based on chemical pre-activation and enzymatic digestion of wool. The effect of mentioned compounds on cell viability of squamous carcinoma cell line and healthy keratinocytes was evaluated. The obtained data show a significantly stronger effect of selected wool hydrolysates compared to commercial compounds (p<0.05) on viability of cells. The wool hydrolysates decreased squamous cell carcinoma cells viability by up to 67% comparing to untreated cells. These results indicate bioactive properties of wool hydrolysates, which affect the viability of squamous carcinoma cells and decrease their number. We hypothesize that these agents may be used topically for treatment of transformed keratinocytes in actinic keratosis and invasive squamous skin cancer in humans.

## Introduction

Squamous cell carcinoma (SCC) is an epithelial malignancy involving many anatomical sites such as: skin, lips, mouth, esophagus, lungs, urinary tract, prostate, vagina, and cervix [1]. Depending on the location, symptoms and treatments can vary. Cutaneous squamous cell carcinoma derives from keratinocyte of spinous layer of the epidermis possess the most important structural elements of keratinocyte such as intermediate filaments and cytokeratins of type 1, 5, 10 and 14 [2]. Cutaneous squamous cell carcinoma (cSCC) is the second most common type of skin cancer worldwide and usually develops on sun-exposed skin areas [3]. Other risk factors besides UV-radiation are: exposure to carcinogenic chemicals (such as coal tar, petroleum oils, arsenic and soot), chronic skin ulceration and immunosuppressive medication in transplant patients [4, 5]. Squamous cell carcinoma is characterized by aneuploidy and deletions of several chromosomes (3p, 9q, 9p,13q, 17p, 17q) and P53 mutations [5]. Despite the generally good prognosis of cSCC, the metastatic SCC is difficult to treat and can be lethal [6]. Low-risk cSCCs have a high cure rate when treated with excision followed by histopathological analysis, electrodessication and curettage or cryosurgery [7]. For invasive cSCC surgical excision or Mohs micrographic surgery are the most appropriate and effective treatment modalities. Radiation therapy can be used as primary treatment for lesions that cannot be surgically excised [4]. Metastatic cSCC can be responsive to some chemotherapeutic agents e.g. cisplatin as a single agent or in combination with 5-fluorouracil (5-FU) [8]. EGFR inhibitors such as cetuximab or erlotinib should be discussed as second line treatment after chemotherapy failure and disease progression [9].

Squamous cell carcinoma can develop from precancerous lesions such as erythroplasia of Queyrat, Bowen’s disease, chronic ulcers and post-radiation scars. Actinic keratosis (AK) is the most common potential precursor of squamous cell carcinoma induced by UV. It is a common skin condition in fair-skinned adults worldwide and regarded as marker of increased risk for non-melanoma skin cancer [10, 11, 12]. Topical pharmacological agents such as 5-FU, diclofenac in hyaluronic acid and ingenol mebutate are effective medications for AK treatment [13–15].

5-FU is an antimetabolite, interfering with DNA synthesis, leading to decrease in cell proliferation and induction of cell death [16]. Ingenol mebutate, a protein kinase C inhibitor, causing cell necrosis (in a few hours), and inducing an inflammatory response (within few days). It has been used for many years to treat different skin conditions, e.g. viral warts, actinic keratosis and tumors [11, 17]. Diclofenac is a nonsteroidal anti-inflammatory drug that reduces the production of prostaglandins through inhibition of cyclooxygenase 2 (COX-2) [18]. It also initiates apoptosis through activation of bcl-2 and caspase 8 [19]. There are new studies about effectiveness of diclofenac in AK treatment [20–22].

It is known the biodegradation products of hair and wool may possess bioprotective properties, which supplement their physical protective function. Markowicz *et al*. in his study showed the anticancer properties of peptide fragments of human hair proteins in human melanoma cell lines [23]. In our study, we show that hydrolysates derived from wool also may have anticancer properties. For such evaluation, we obtained several peptides and proteins mixtures from enzyme-digested wool and tested their effect on human epithelial skin cell lines *in vitro* (squamous carcinoma cell line and healthy keratinocytes). Also commercially available anticancer compounds (5-FU, diclofenac sodium salt, ingenol mebutate were tested on SCC and healthy epidermal keratinocytes in comparison to wool hydrolysates to evaluate their effects as an established factors in SCC prevention and skin malignancies treatment.

## Materials and methods

### Preparation of wool hydrolysates

Process of wool degradation was based on Lipkowski methods described earlier [24, 25]. Wool was suspended in NaOH solution for at least 1 hour at room temperature (21°C) for pre-activation. Next, wool was filtered off and washed twice with water. Then, it was suspended in water which was acidified to pH = 2 with pepsin and reaction was continued with mixing for 24 hours. After the pepsin digestion, the solution was heated to 80°C to inactivate pepsin, and then cooled to approx. 40°C, filtered and washed with water (S1 Fig). The resulting raw water fraction was filtered, separated, frozen and lyophilized. Some of the hydrolysates were subjected to extraction with different solvents to extract hydrolysates subfractions. Due to the relevant physicochemical properties of hydrolysates, four of them were chosen for further experiments: MR1, MR2, MR3, MR4. The separation process was based on the assumption that after extracting an aqueous solution of hydrolysate mixture with diethyl ether, two separate fractions—the upper fraction containing compounds soluble in diethyl ether (more hydrophobic) and the bottom fraction containing compounds soluble in water (more hydrophilic) will be obtained. Afterwards, both fractions were lyophilized and the probes were subjected to check their activity. The probes MR1, MR2 and MR4 represent aqueous fractions and MR3 the upper fraction obtained after extraction process.

### Lowry assay

Lyophilized filtrates of hydrolysates (raw) and fractions after extraction were analyzed. Quantification of total proteins and peptides content in wool hydrolysates was performed using Micro Lowry Onishi & Barr Modification assay (detailed information are present in S1 File).

### Commercially available compounds

Three commercially available compounds were tested in this study: 5-FU, ingenol mebutate and diclofenac sodium salt (Sigma Aldrich, Poland). The smallest active molar concentrations were used in experiments. 5-FU was used in this study as a control anticancer drug.

### Cell culture

Cell culture was performed on the following cell lines: transformed human keratinocytes SCC-25 and human epidermal keratinocytes HEKa. Human squamous cell carcinoma cell line SCC-25 was purchased from DSMZ (cat. no. ACC 617). Human epidermal keratinocytes HEKa were purchased from CellSystems (cat. no. FC-0025). SCC-25 cells were cultured in mixture of Ham's F12/Dulbecco's MEM (at 1:1) (Sigma Aldrich, Poland) with (v/v) 20% h.i. FBS, 2 mM L-glutamine, 1 mM sodium pyruvate and 1% penicillin-streptomycin (Sigma Aldrich, Poland). Human keratinocytes HEKa were cultured in EpiLife medium (Gibco) with Epi Life supplement (Gibco) and 1% antibiotic (Sigma Aldrich, Poland). All cells were maintained in a humidified incubator with 5% CO_2_ at 37°C.

### Isolation of epidermal keratinocytes (sHEK)

Keratinocytes were isolated from normal skin samples obtained from healthy patients after the breast reduction surgery. This method was approved by the Bioethics Committee of Medical University of Warsaw, Poland (KB/283/2013). Keratinocytes were isolated using the enzymatic tissue dissociation procedure. Fragments of human skin after removal of adipose tissue and rinsing with PBS with 1% antibiotics were incubated in dispase II (Sigma Aldrich, Poland) overnight at 4°C, after which the epidermis was separated from the dermis mechanically. The separated epidermis was digested for a few minutes in trypsin with 0.05% EDTA. Trypsin was inactivated with addition of DMEM with 10% (v/v) FBS. Next, cell suspension was filtered with 40 μm strainer (Sigma, Steinheim, Germany) and centrifuged for 5 minutes at 1200 rpm. Supernatant was discarded. The cell pellet was resuspended in DMEM and 10% (v/v) FBS with 1% antibiotic-antimycotic solution (Sigma Aldrich, Poland). Human isolated keratinocytes from skin (sHEK) were cultured in EpiLife medium (Gibco) with Epi Life supplement (Gibco) and 1% antibiotic (Sigma Aldrich, Poland). Cells were maintained at 37°C in a humidifying atmosphere of 5% CO_2_. The culture medium was changed after 24h.

## Immunocytochemistry study

### Characterization of isolated keratinocytes

For immunofluorescence analysis, isolated sHEK cells were cultured on glass cover slips (Ø12mm) in 24-well plate. Cells were washed with PBS and fixed with 4% paraformaldehyde in PBS for 20 min at room temperature. Next, cells were blocked in 10% goat serum in 0.25% Triton X-100 (in PBS) for 60 min at room temperature. Then cells were incubated overnight at 4°C with mouse monoclonal antibodies anti-Cytokerain-14 (sc-53253, Santa Cruz) diluted in a blocking mixture. Next, cells were washed with PBS, and incubated for an additional 60 min at room temperature with AlexaFluor546-conjugated goat anti-mouse IgG (A11003, Invitrogen) at 1:500 dilution. Cells nuclei were stained with Hoechst33342 dye. Samples were closed with Fluorescent mounting medium (Dako) and visualized using an Axioscope microscope with AxioCamMRC5 camera and confocal LSM 510 system (Carl Zeiss GmBH, Jena, Germany).

### Expression of Ki-67

All cell lines were cultured on glass cover slips (Ø12mm) in 24-well plate. Cells were washed three times with PBS and fixed with 4% PFA in PBS for 20 min at room temperature. Next, cells were washed three times with PBS and blocked in 10% goat serum in 0.25% Triton X-100 (in PBS) for 60 min at room temperature. Then cells were incubated overnight at 4°C with anti-Ki-67 antibodies (Novocastra, clone MM1) diluted in a blocking mixture. Next, cells were washed three times with PBS, and incubated for an additional 60 min at room temperature with AlexaFluor546-conjugated goat anti-mouse IgG (A11003, Invitrogen) at 1:500 dilution. Cells nuclei were stained with Hoechst33342 dye. Next, cells were washed with PBS and closed with Fluorescent Mounting Medium (S3023, Dako).

## Cell viability assays

Cell viability was measured with CellTiter 96 Aqueous One Solution Cell Proliferation Assay (Promega) according to manufacturer instructions. SCC-25 were seeded in 96-well plates at density of 2x10^3^, HEKa and sHEK cells were seeded in 96-well plates at density of 3x10^3^ cells/well. After 24h of incubation, hydrolysates (at final concentrations of 0.1%, 0.5% and 1%) or commercially available compounds (at final concentrations of 25 μM, 50 μM and 100 μM) were added to the cells. 10% DMSO in sterile ddH_2_O was used as a solvent in all experiments, including the control wells. After 24h of incubation medium was changed to remove any insoluble particles and 20 μl of MTS solution was added into each well and incubated for 1h at 37°C, 5% CO_2_. The intensity of absorbance was measured at 490 nm using a Cytation3 microplate reader. All experiments were performed in triplicates. There were no pick coincidence in absorbance of salt MTS and tested compounds (S2a and S2b Fig).

In selected experiments, the influence on cell viability was confirmed by trypan blue exclusion test. Cells were seated in 24-well plates at density of 10000 per well. Plates were incubated overnight. Next hydrolysates (at final concentrations of 0.1%, 0.5% and 1%) and 5-FU, ingenol mebutate and diclofenac sodium salt (at final concentrations of 25 μM, 50 μM and 100 μM) were added to wells. Cells were harvested after 24 h using 0.25% trypsin with EDTA. Harvested cells were centrifuged, resuspended in fresh media, stained with trypan blue (1:1 ratio) and counted using EVE Automated Cell Counte (NANOENTEK USA INC). All experiments were performed in triplicates.

## Statistical analysis

Prior the analysis, the dataset has been checked with Dixon test to remove outliers. Breakdown and one-way ANOVA has been applied to test the hypotheses that different concentrations of selected compounds are significantly changing the cell viability. Newman-Keuls post hoc test (p = 0.05) was used to check differences in absorbance of investigated compounds at various concentrations within three cell lines.

Comparison between different cell lines at the same concentrations have been analyzed by means of t-test (p = 0.05) and crosschecked with nonparametric Mann Whitney test (p = 0.05).

The results are presented as box plots. Asterisks indicate the significance of difference. However one has to notice that due to the testing of differences between all variants, different star colors were used. The variants differ significantly only if the asterisk color is various. Black asterisk means that selected variant differs with all the others. In contrast, the same color indicates that difference is statistically irrelevant. Moreover, number of asterisks (*; ** or ***) refers to p < 0.05; p < 0.01 and p < 0.005, respectively.

All analyses were performed in Statistica DataMiner 7 (Statsoft, Poland).

## Results

### Peptides and proteins content

The quantitative content of peptides and proteins in wool hydrolysates using Lowry assay showed that each hydrolysate contained more than 66% peptides and proteins. The highest content of peptides and proteins (85%) was noticed in MR4 sample (Table 1). In most samples of hydrolysates, difficulties with solubility were observed. Thus, only low percentage concentrations of four selected, most soluble hydrolysates (MR1-MR4) were tested *in vitro*.

**Table 1:** Total peptides and proteins content in wool hydrolysates.

*Hydrolysate*	*% of proteins/peptides* ± *cv*
MR1	66±0.4
MR2	66±1.1
MR3	73±0.9
MR4	85±0.5
MR5	80±0.4
MR6	79±0.9
MR7	77±1.5
MR8	70±0.6

### Morphology of isolated cells

Cells isolated from human skin exhibited typical epithelioid morphology (S3a Fig). Isolated keratinocytes did not require a fibroblast feeder layer to support their growth. Cells were not immortalized and after third passage cell populations became more heterogeneous, displaying outstretched shape and enlarged cytoplasm with ultrastructural alterations (S3b Fig). Although nuclei did not change in size, they presented irregular shapes. Also in the older passages, cell division was inhibited or was very slow. Isolated skin keratinocytes (sHEK) stained positively with anti-cytokeratin 14 (CK14) antibody (S3c Fig).

### Anti-Ki-67 antibodies staining

Untreated cells stained positively with anti-Ki-67. The average value of proliferation index Ki-67 shown as mean ± SD in SCC-25 cell line was 62.63±13,62%, in isolated sHEK cells was 35,5±9,29% and for HEKa was 53,00±16,52% (S4 Fig). Obtained results indicate differences in cell proliferation rate between cell lines, and isolated keratinocytes sHEK showed the lowest percentage of proliferating cells.

### Cell viability

**Fig 1:**
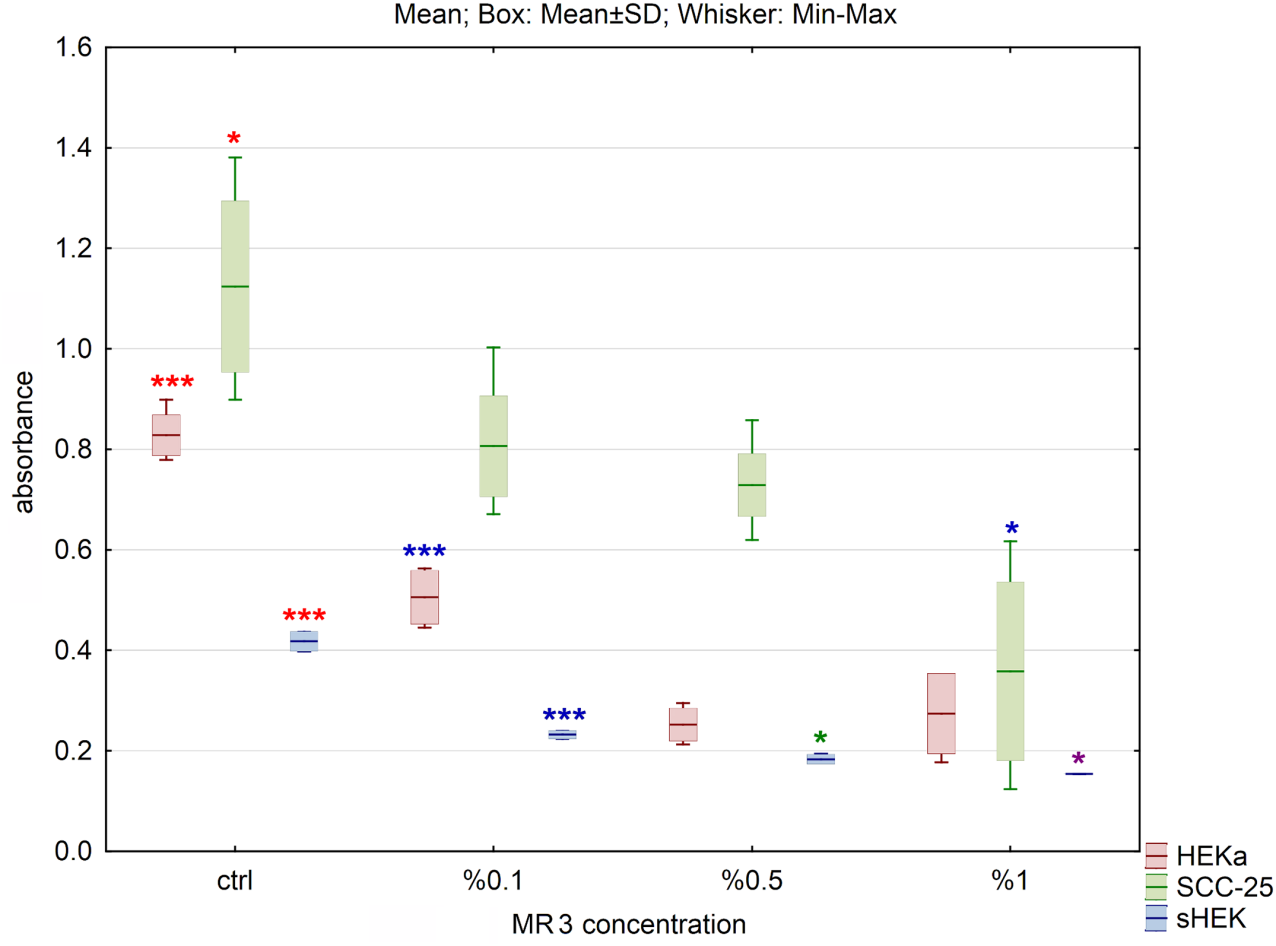
The effect of MR3 hydrolysate on cell viability of three cell lines after 24h of incubation. The results are presented as box plots. Asterisks indicate the significance of difference. However one has to notice that due to the testing of differences between all variants, different star colors were used. The variants differ significantly only if the asterisk color is various. Black asterisk means that selected variant differs with all the others. In contrast, the same color indicates that difference is statistically irrelevant. Moreover, number of asterisks (*; ** or ***) refers to p < 0.05; p < 0.01 and p < 0.005, respectively.

**Fig 2:**
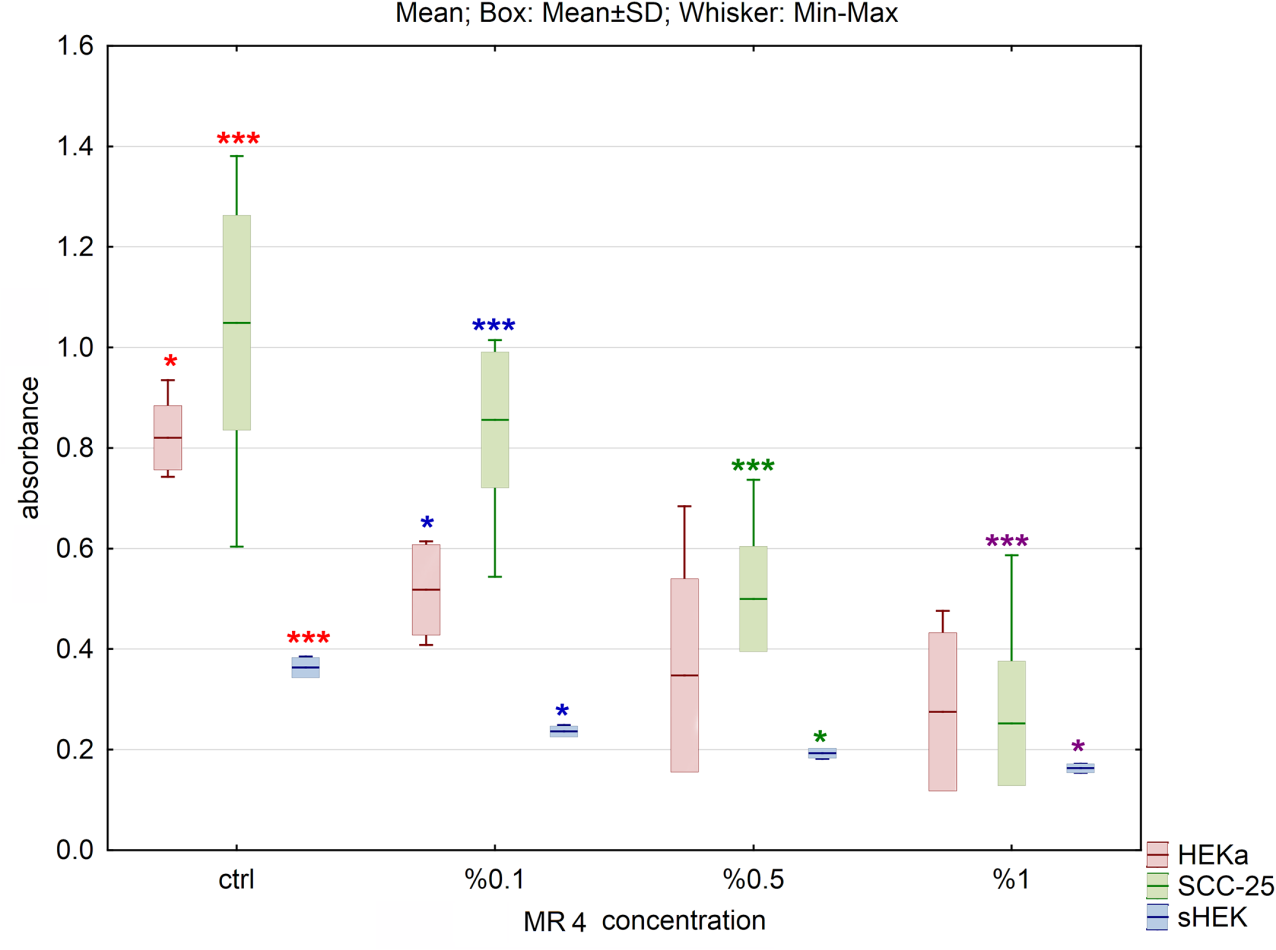
The effect of MR4 hydrolysate on cell viability of three cell lines after 24h of incubation. The results are presented as box plots. Asterisks indicate the significance of difference. However one has to notice that due to the testing of differences between all variants, different star colors were used. The variants differ significantly only if the asterisk color is various. Black asterisk means that selected variant differs with all the others. In contrast, the same color indicates that difference is statistically irrelevant. Moreover, number of asterisks (*; ** or ***) refers to p < 0.05; p < 0.01 and p < 0.005, respectively.

**Fig 3:**
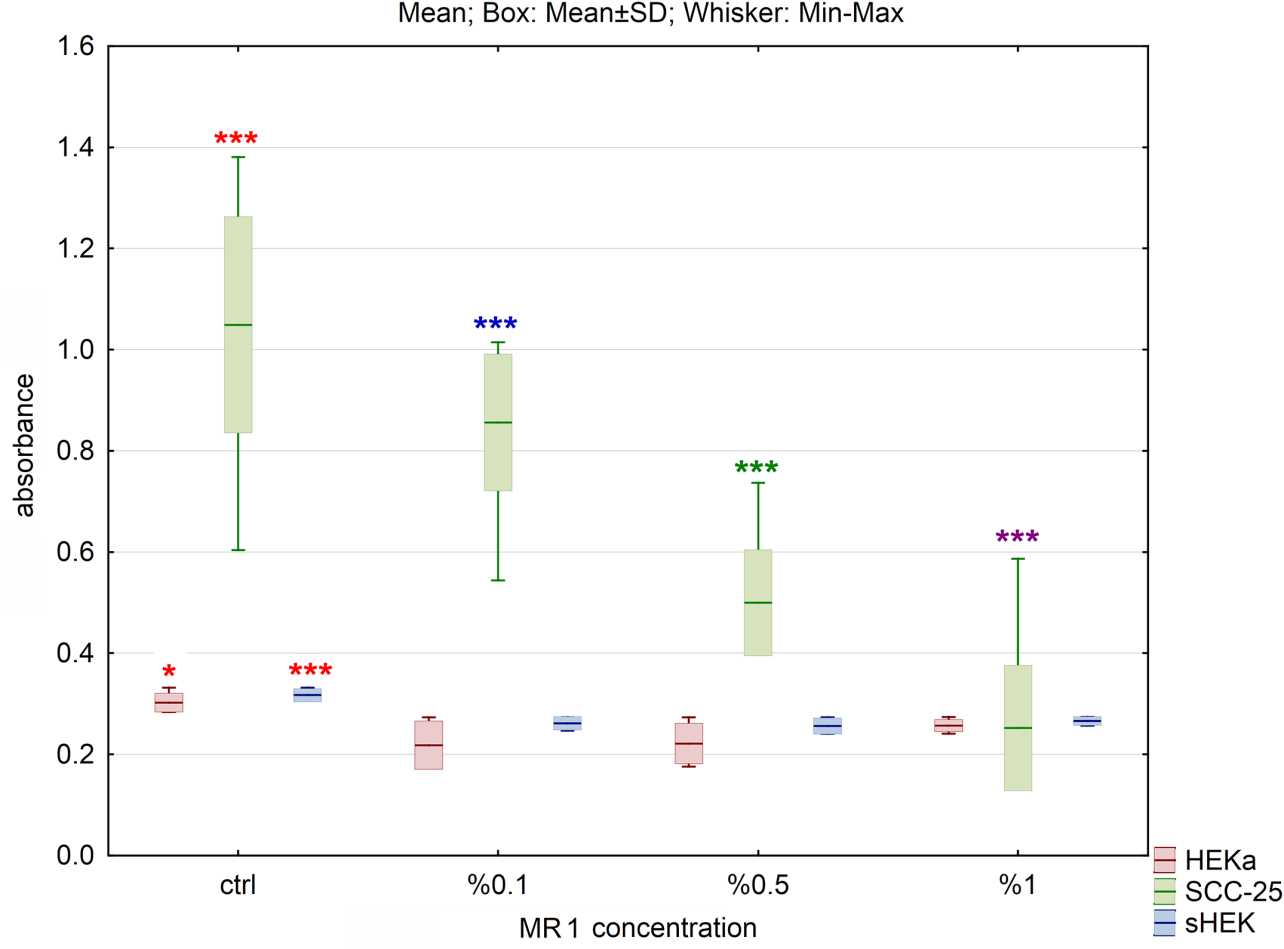
The effect of MR1 hydrolysate on cell viability of three cell lines after 24h of incubation. The results are presented as box plots. Asterisks indicate the significance of difference. However one has to notice that due to the testing of differences between all variants, different star colors were used. The variants differ significantly only if the asterisk color is various. Black asterisk means that selected variant differs with all the others. In contrast, the same color indicates that difference is statistically irrelevant. Moreover, number of asterisks (*; ** or ***) refers to p < 0.05; p < 0.01 and p < 0.005, respectively.

**Fig 4:**
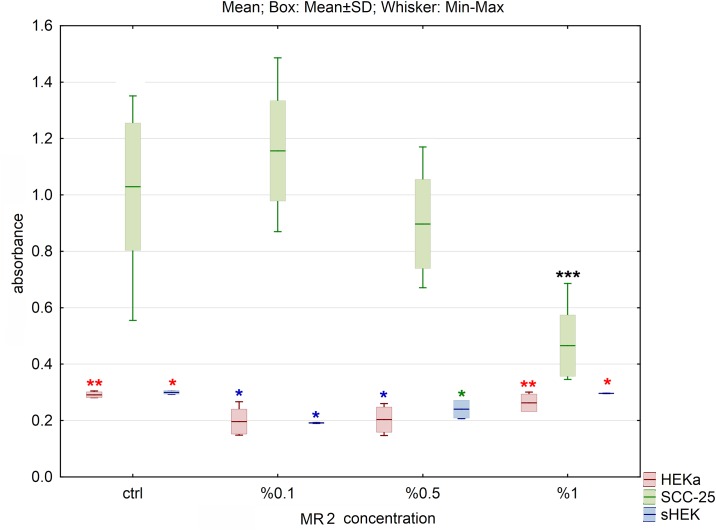
The effect of MR2 hydrolysate on cell viability of three cell lines after 24h of incubation. The results are presented as box plots. Asterisks indicate the significance of difference. However one has to notice that due to the testing of differences between all variants, different star colors were used. The variants differ significantly only if the asterisk color is various. Black asterisk means that selected variant differs with all the others. In contrast, the same color indicates that difference is statistically irrelevant. Moreover, number of asterisks (*; ** or ***) refers to p < 0.05; p < 0.01 and p < 0.005, respectively.

Cell viability, presented on graphs as an absorbance, was determined by the MTS assay and calculated by comparison to the control. We have not observed decrease in cell viability treated with 10% DMSO in water. The obtained data showed decrease in cell viability in HEKa, sHEK and SCC-25 cells treated with selected hydrolysates. For all tested cell lines, the most significant decline of cell viability was observed for MR3 and MR4 hydrolysates (Figs 1 and 2), while for MR1 and MR2 the difference between control and hydrolysates was less significant (Figs 3 and 4). Differences of absorbance of MR3 within HEKa, SCC-25 and sHEK cell lines were significant with p values smaller than 0.001, 0.05 and 0.05, respectively. p values for MR4 were smaller than 0.05, 0.005 and 0.005 within HEKa, SCC-25 and sHEK cell lines, respectively. Viability of SCC-25 and sHEK cells treated with MR4 decreased significantly in a dose dependent manner with increasing concentration of MR4 (p<0.005). There were no significant changes in viability of HEKa cells treated with the highest concentrations of MR4 (Fig 2). Hydrolysate MR4 caused reduction in cell viability for SCC-25 up to 70% to untreated control cells and up to 40% and 60% for isolated sHEK and HEKa keratinocytes respectively.

Addition of MR1 caused significant decrease of cell viability in case of SCC-25 cells for all hydrolysate concentrations used (p<0.005). In the case of sHEK and HEKa cells, treatment with MR1 caused significant decrease of cell viability compared to control (p<0.005 and p<0.05, respectively). No change between variants with different concentrations was observed (Fig 3).

The smallest impact on cell viability was observed for MR2 (Fig 4). For SCC-25 cells only the highest concentration caused significant change of cell viability (p<0.01). For HEKa cell line the smallest but still significant changes in cell viability were observed (p<0.05). Addition of 0.1% suspension of MR2 induced significant decrease of cell viability in sHEK (p<0.05), but at concentration of 0.5% increase of cell viability was observed. Intensity of absorbance at 1% was similar to absorbance of the control for examined keratinocytes.

Moreover, comparison between different cell lines treated with the same concentrations of examined compounds was also analyzed. The absorbance measured in SCC-25 and both, in sHEK and HEKa cells was significantly different for all investigated compounds in all used concentrations.

The trypan blue dye exclusion assay was used to detect alive cells that were reported as the percentage of viable vs total number of counted cells. Results obtained by trypan blue staining were similar to MTS results and showed significant decrease in number of viable SCC-25 cells compared to untreated cells (S4 and S5 Figs). The relative percent of viable cells decreased to even 20% in wells treated with hydrolysates.

**Fig 5:**
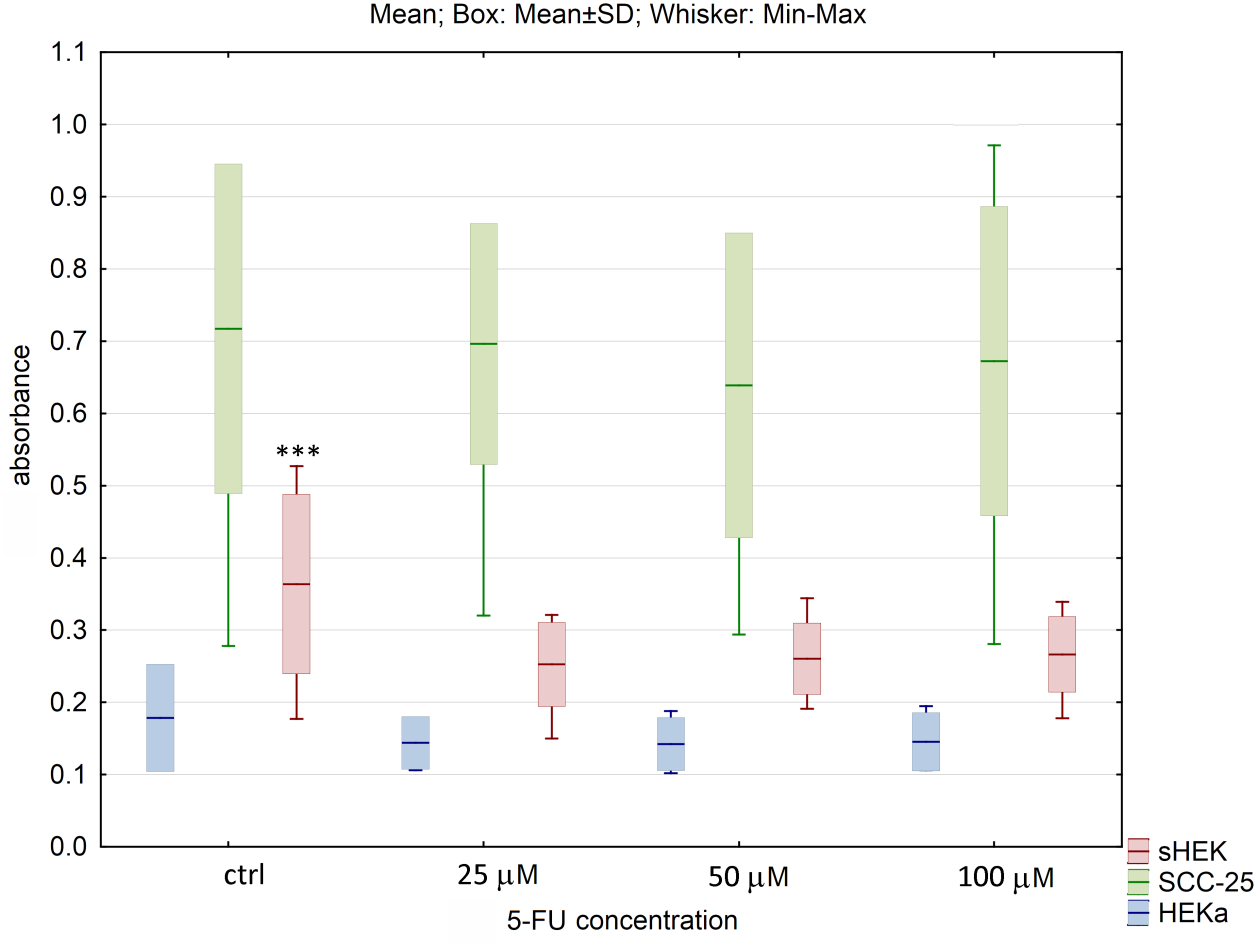
The effect of 5-fluorouracil on cell viability of three cell lines after 24h of incubation. The results are presented as box plots. Asterisks indicate the significance of difference. However one has to notice that due to the testing of differences between all variants, different star colors were used. The variants differ significantly only if the asterisk color is various. Black asterisk means that selected variant differs with all the others. In contrast, the same color indicates that difference is statistically irrelevant. Moreover, number of asterisks (*; ** or ***) refers to p < 0.05; p < 0.01 and p < 0.005, respectively.

**Fig 6:**
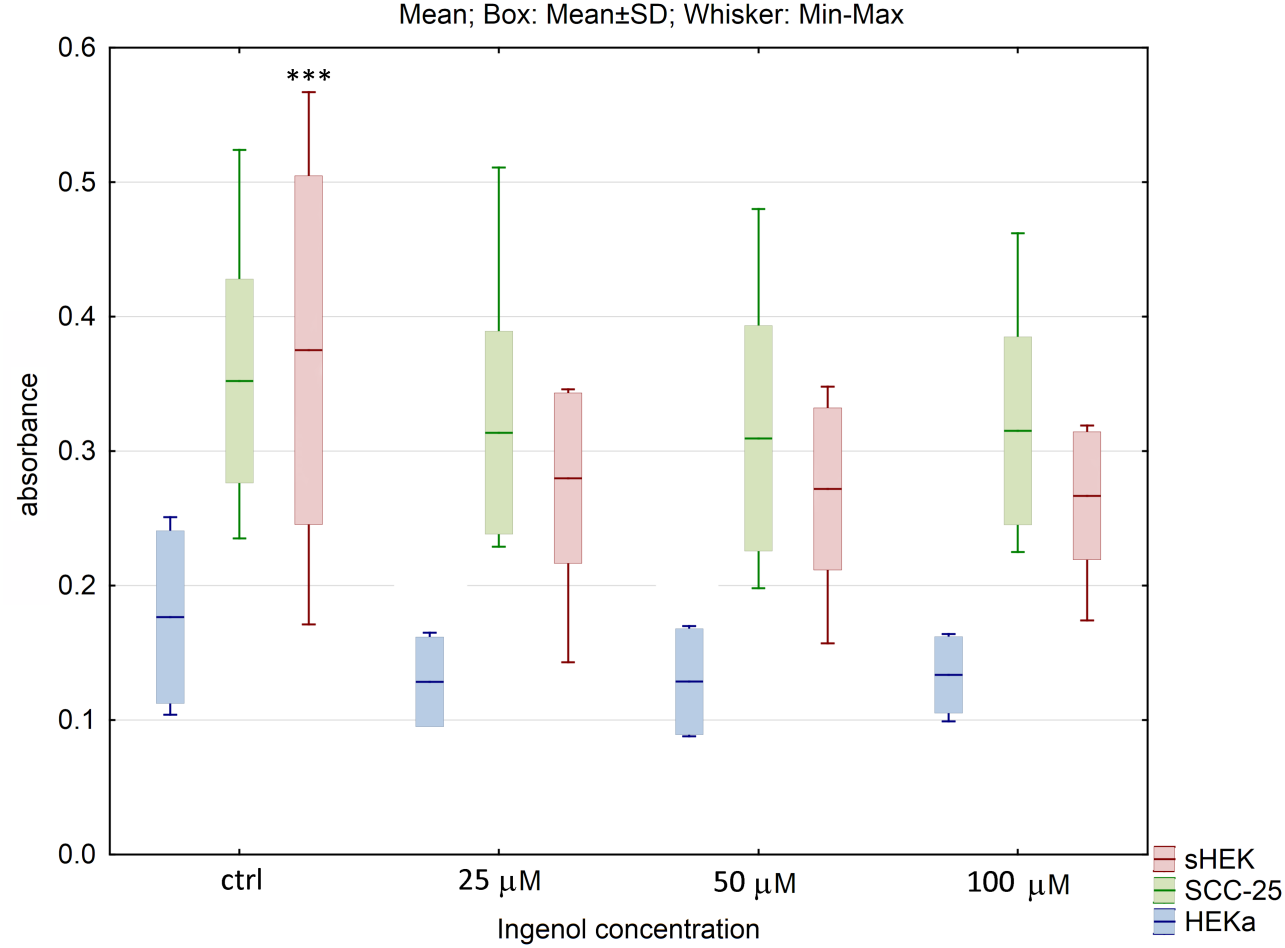
The effect of ingenol mebutate on cell viability of three cell lines after 24h of incubation. The results are presented as box plots. Asterisks indicate the significance of difference. However one has to notice that due to the testing of differences between all variants, different star colors were used. The variants differ significantly only if the asterisk color is various. Black asterisk means that selected variant differs with all the others. In contrast, the same color indicates that difference is statistically irrelevant. Moreover, number of asterisks (*; ** or ***) refers to p < 0.05; p < 0.01 and p < 0.005, respectively.

Also, the effect of commercially available anticancer compounds on cell viability was evaluated. For 5-FU and ingenol mebutate at all concentrations used, no statistically significant changes in viability of SCC-25 and HEKa cells were observed. For isolated sHEK cells, significant difference between control and the lowest concentration of these compounds (25 μM) was observed (p<0.005) (Figs 5 and 6). Furthermore, the number of dead cells for SCC-25 and healthy keratinocytes, was relatively low and did not significantly differ between controls and treated cells.

**Fig 7:**
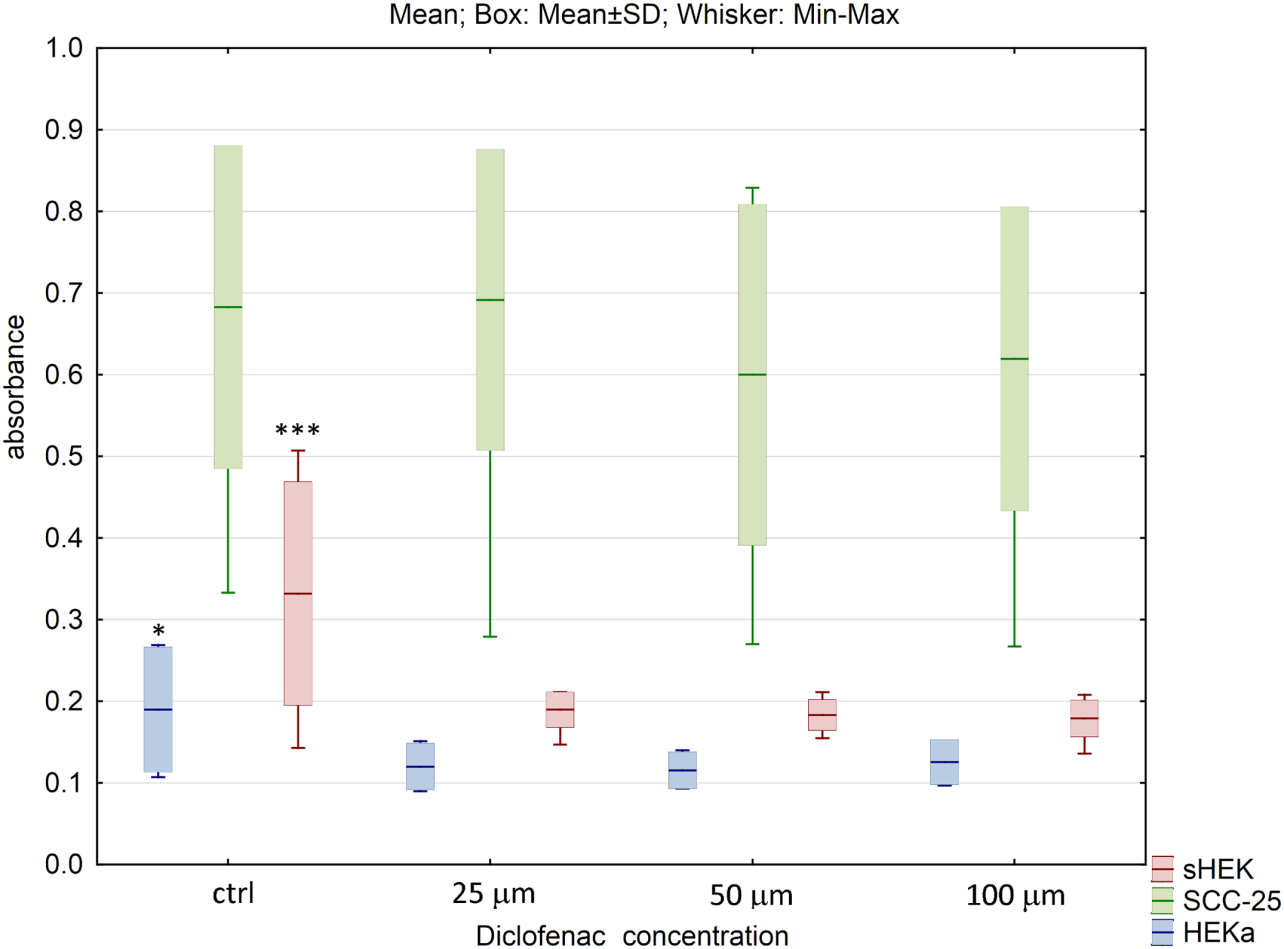
The effect of diclofenac sodium salt on cell viability of three cell lines after 24h of incubation. The results are presented as box plots. Asterisks indicate the significance of difference. However one has to notice that due to the testing of differences between all variants, different star colors were used. The variants differ significantly only if the asterisk color is various. Black asterisk means that selected variant differs with all the others. In contrast, the same color indicates that difference is statistically irrelevant. Moreover, number of asterisks (*; ** or ***) refers to p < 0.05; p < 0.01 and p < 0.005, respectively.

The similar effects were observed for diclofenac sodium salt, where in case of SCC-25 no cell viability changes were noticed, but for HEKa and sHEK cell lines, diclofenac addition at the lowest concentration of 25 μM induced significant decrease of cell viability (p<0.05 and p<0.005 in HEKa and sHEK, respectively) (Fig 7).

**Fig 8:**
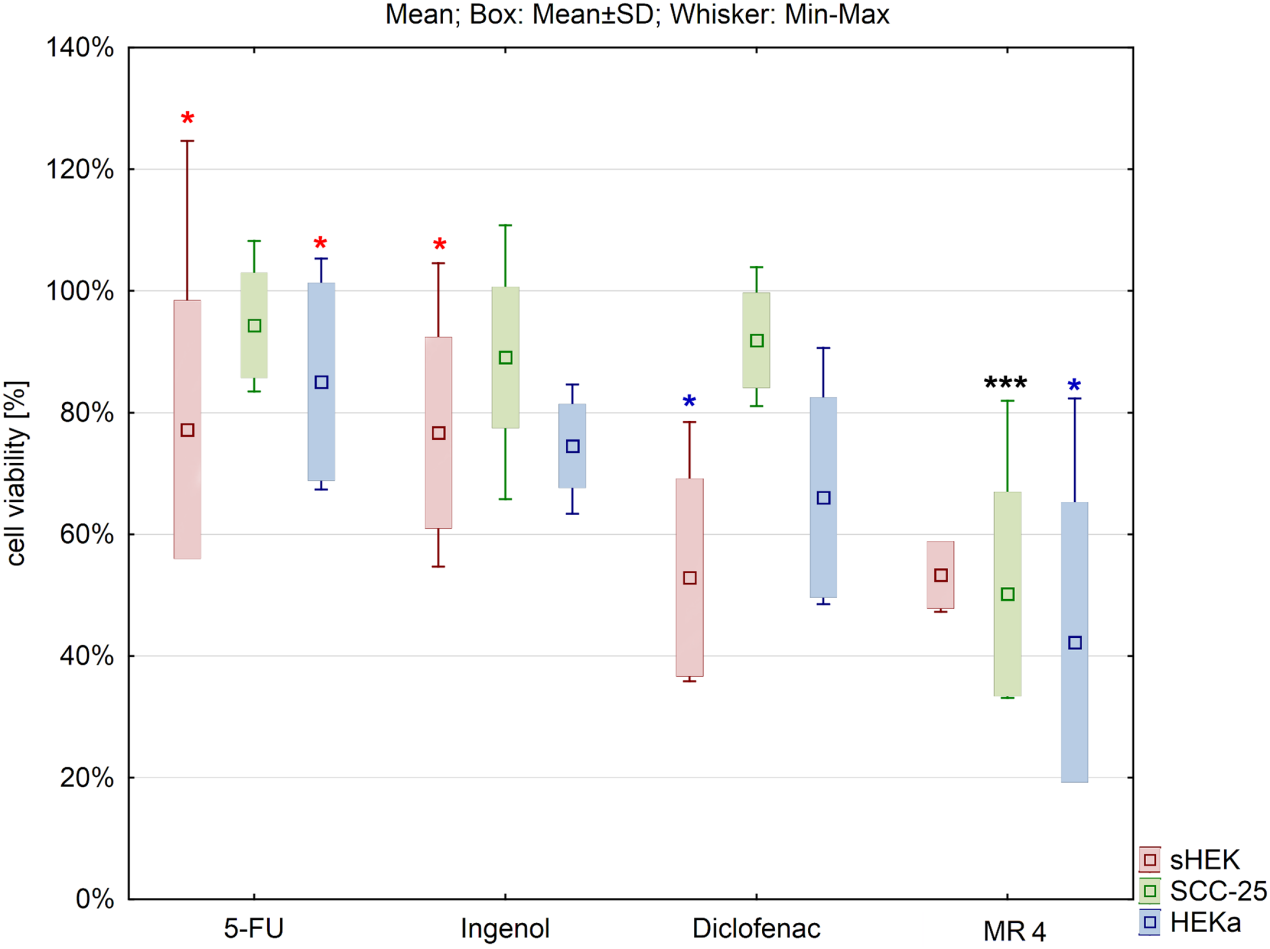
Comparison of the effect of selected compounds on cell viability of three cell lines in the middle concentrations (for MR4 0.5% and 50 μM for the commercial compounds). The results were expressed as a percentage of the viability of treated cells to untreated ones. The results are presented as box plots. Asterisks indicate the significance of difference. However one has to notice that due to the testing of differences between all variants, different star colors were used. The variants differ significantly only if the asterisk color is various. Black asterisk means that selected variant differs with all the others. In contrast, the same color indicates that difference is statistically irrelevant. Moreover, number of asterisks (*; ** or ***) refers to p < 0.05; p < 0.01 and p < 0.005, respectively.

Differences in cell viability between cells treated with 0.5% MR4 and 50 μM of 5-FU, diclofenac sodium salt and ingenol mebutate were also evaluated (Fig 8). The cell viability was significantly lower in case of hydrolysate addition comparing to commercial substances. Only for sHEK cell line there were no changes in cell viability when diclofenac sodium salt was added in the concentration of 50 μM compared to the effect of 0,5% of hydrolysate MR4.

## Discussion

In the last decades several studies reported reduced cancer risk associated with natural substances consumption [26, 27]. There are studies suggesting a possible antiproliferative effects of natural products, mostly plant extracts, in prevention and treatment of skin squamous cell carcinoma [28–30]. Research on the anticancer effects of hair hydrolysates were initiated by A.W. Lipkowski from our team, and the first tested cutaneous cells were melanoma cells [23]. The authors showed the antiproliferative effects of hair hydrolysates at relatively high concentrations (0.1% m/v). The preliminary results from this study suggest, that obtained wool hydrolysates can be used as a potential source of anticancer drugs.

Studies about isolation, characterization and cytotoxicity of biofunctional keratin particles extracted from wool were performed in the recent few years. J. Zhang et al. examined the nontoxicity and biocompatibility of wool fibers hydrolyzed in the acidic environment using hydrochloric acid [31]. The results showed that keratin particles KP3 and KP5 (at concentrations of 0.1 mg/ml and 1 mg/ml, respectively), possessed expedient impact on cell viability of fibroblasts cell line. These results demonstrated that both keratin particles could be tailored from wool hydrolyzed solution and had potential future application as biomaterials for wound healing and drug delivery. Another study by Li et al. revealed that 0.5 mg/ml of wool hydrolysate PP5.55 had no negative effect on fibroblasts viability [32]. Although this study indicated the absence of toxicity in wool polypeptide and supported its safe use as a food ingredient or drug carrier, their fate, kinetics, clearance, metabolism and immune response are still not fully understood.

In our study, we aimed to estimate the potential antitumor effect of wool hydrolysates, which are easy accessible and relatively inexpensive natural products. We tested a highly metastatic cancer cell line SCC-25, and two normal keratinocyte cell lines. One of them was isolated from the human healthy skin. The latest study from the literature showed Ki-67 immunostaining proliferative index values to be between 15–84% in cutaneous squamous cell carcinoma lines, with the highest expression reported in poorly differentiated tumors [33]. In our study the average Ki-67 proliferative index for examined SCC-25 cell line was 62%.

In order to obtain fractions rich in protein and peptide from the wool, three methods—acidic hydrolysis, enzymatic hydrolysis and alkaline hydrolysis can be used to hydrolyze wool fibers. In our study, wool hydrolysates derived from enzymatic hydrolysis of wool, possessed the best physico-chemical characteristics, proved to be more effective in decreasing viability of cancer cells than healthy ones. The hydrolysates inhibited cell proliferation in a dose- dependent manner which indicates their bioactive and antiproliferative properties.

**Fig 9:**
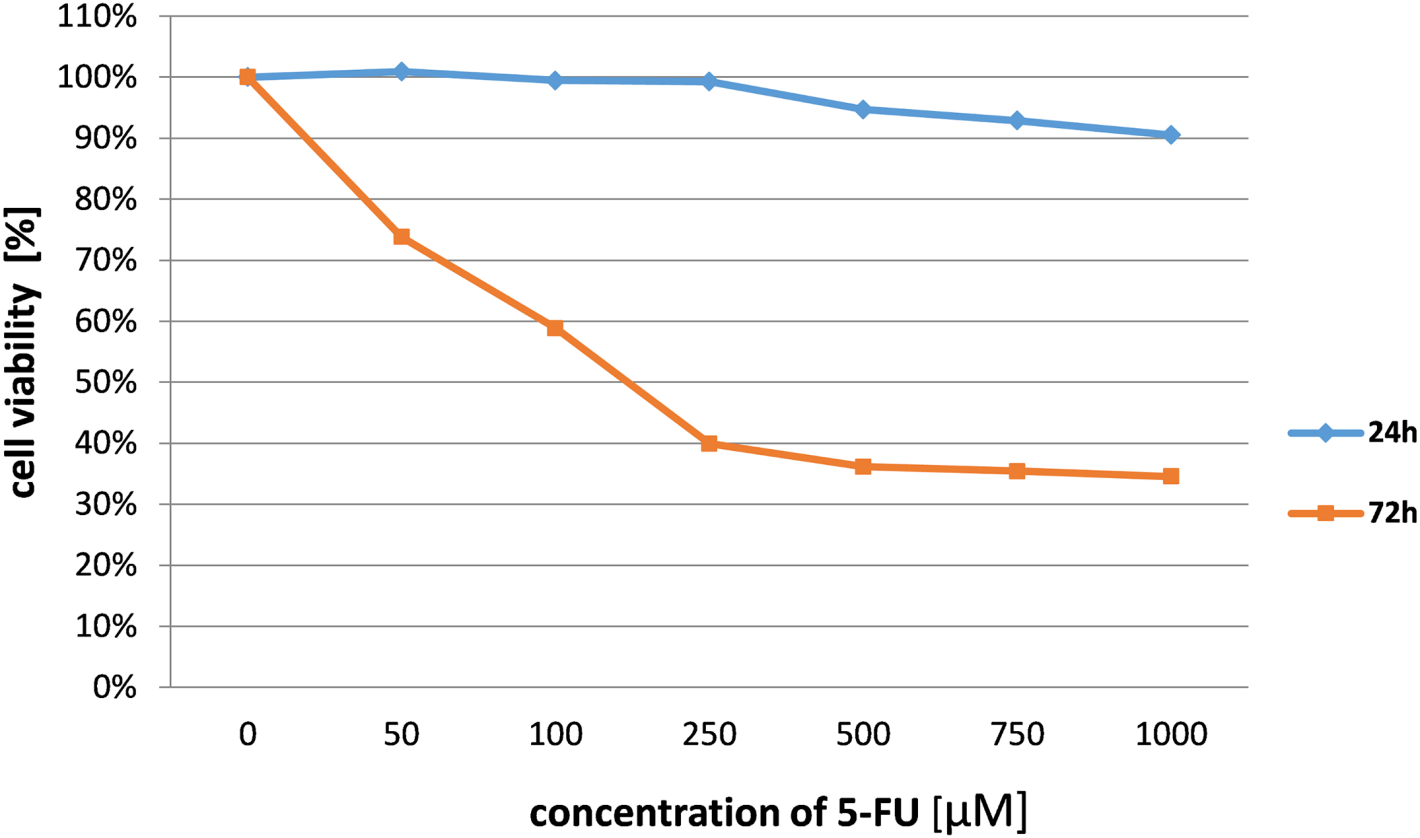
The effect of 5-fluorouracil on cell viability of SCC-25 cell line after 24h and 72h of incubation.

The rising incidence of invasive squamous cell carcinoma developing from actinic keratosis leads to necessity of searching for new agents which inhibits transformed keratinocytes proliferation. We tested the cytotoxic effect of animal wool enzymatic hydrolysis products and commercially available compounds on a squamous carcinoma cell line and adult human epidermal keratinocytes. Our results indicate that wool hydrolysates express a significant effect on viability of SCC cells while having minimal effect on healthy keratinocytes. Those differences were statistically significant and were not observed in the majority of cases of all investigated commercially available anticancer compounds (5-FU, ingenol mebutate, diclofenac sodium salt). The data presented in the main text showed results obtained only after 24 h incubation. We did not present data obtained after 72 h of treatment. While, 24 h incubation with 5-FU does not affect cell growth, extended treatment resulted in significant decrease of cell viability as shown in Fig 9. Higher activity of 5-FU after 72 h might be attributed to its mechanism of action, interference in S phase of cell cycle. Individual cells might be in different phase of the cycle, therefore longer incubation is needed to observe changes in cell viability. Since we are presenting data after 24 h for hydrolysates, we decided to do the same for the referring drugs.

For the first time in the literature, we present novel experimental results about the antiproliferative selective effects of wool hydrolysates against squamous carcinoma cells. The precise mechanism of action of tested wool hydrolysates is still unknown, because it is a mixture of several proteins and peptides. Therefore, further experiments are necessary to identify and isolate active compounds with recognition of the mechanism of wool hydrolysates action, which may lead to elaboration of new anticancer drug.

The most common precancerous form of SCC is actinic keratosis connected to partial transformation of keratinocyte due to chronic and cumulative UV exposure and DNA damage [34]. AK with time can progress into complete transformation, loss of keratinocyte differentiation, and ability to invade the dermis [35, 36]. At this moment there is no commercially available cell line corresponding to actinic keratosis phase and it is difficult to get them from the sun damaged skin, since this is not preventively excised. But based on our results, we hypothesize that wool hydrolysates may be considered as topical treatment for actinic keratosis to prevent dysplastic keratinocytes from progression and transformation into squamous cell carcinoma, based on their bioactive properties and selectiveness as our *in vitro* results showed.

## Supporting information

S1 Fig Preparation of hydrolysates. (TIF)

S2 Fig Absorbance spectrum of commercial compounds (a) and two hydrolysates (b), as a control group was used dimethyl sulfoxide (DMSO) in water. The peak of absorbance spectrum of used substances do not overlap with MTS/formazan peak absorbance between 450-500nm.
(TIFF)

S3 Fig Isolated sHEK cells under light microscope (a—second passage, b—fourth passage) and stained with CK14 (c). Magnification 100x.
(TIF)

S4 Fig Proliferation index for cell lines SCC-25, sHEK and HEKa after 24h of incubation. Statistical analysis used average values and comparison tests (ANOVA, Kruskal-Wallis/Dunn's Multiple Comparison tests) with Prism (* p<0,05).
(TIF)

S5 Fig Viability of cells treated with hydrolysates and stained with trypan blue after 24h of incubation. Statistical significance: (*) p<0.05, (**) p<0.01, (***) p<0.001.
(TIFF)

S6 Fig Viability of cells treated with commercial compounds and stained with trypan blue after 24h of incubation. Statistical significance: (*) p<0.05, (**) p<0.01, (***) p<0.001.
(TIFF)

S1 File To prepare the calibration curve, bovine serum albumin (BSA) in 0.85% NaCl was used as a standard. 0.85% NaCl served also as a blank. 1 mg of each sample was dissolved in 1 mL of 0.85% NaCl. 200 mL of each sample was transferred to 5 mL tubes followed by addition of 2.2 mL of Biuret reagent. Solution in each tube was stirred immediately and allowed to stand for 10 minutes. Next, 100 uL of Folin & Ciocalteu’s phenol reagent was added, obtained solution stirred and allowed to stand for 30 minutes. The solutions were subsequently transferred to 96-well plate and the absorbance was measured at a wavelength of 750 nm using Cytation3 microplate reader. Each of the sample was tested simultaneously in quadriplicate, and each of the experiments was repeated two times.
(DOCX)

## Abbreviations

5-FU: 5-fluorouracil
AK: actinic keratosis
BSA: bovine serum albumin
CK14: cytokeratin 14 antibody
cSCC: cutaneous squamous cell carcinoma
DMEM: Dulbecco's modified Eagle’s medium
DMSO: dimethyl sulfoxide
DSMZ: Deutsche Sammlung von Mikroorganismen und Zellkulturen GmbH
EDTA: ethylenediaminetetraacetic acid
EGFR: epidermal growth factor receptor
FBS: fetal bovine serum
HEKa: human epidermal keratinocytes adult
Ki-67: proliferation-related antigen
MTS: [3-(4,5-dimethylthiazol-2-yl)-5-(3-carboxymethoxyphenyl)-2-(4-sulfophenyl)-2H-tetrazolium], inner salt
NaCl: sodium chloride
NaOH: sodium hydroxide
PBS: phosphate buffered saline
SCC: squamous cell carcinoma
SCC-25: squamous cell carcinoma cell line
SD: standard deviation
sHEK: skin human epidermal keratinocytes
UV-radiation: ultraviolet radiation
