# Supplementary figures and images for: The effect of wool hydrolysates on squamous cell carcinoma cells in vitro. Possible implications for cancer treatment
Source: PLoS One. 2017 Aug 31;12(8):e0184034. doi: 10.1371/journal.pone.0184034 (PMC5578621; doi:10.1371/journal.pone.0184034)

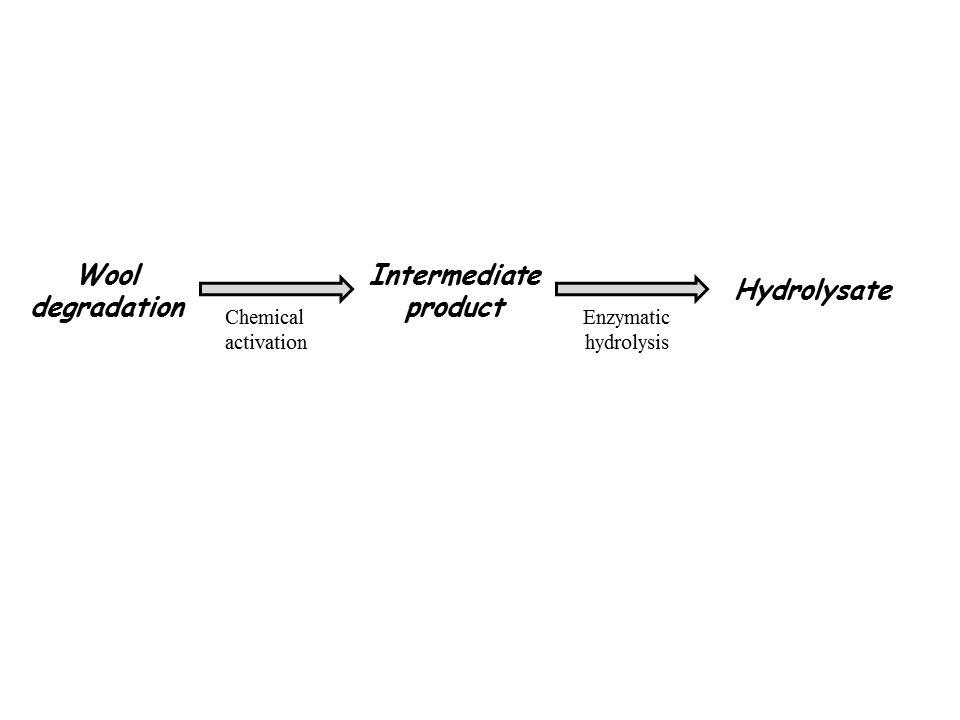

Supplement: S1 Fig — (TIF) [file pone.0184034.s001.tif]

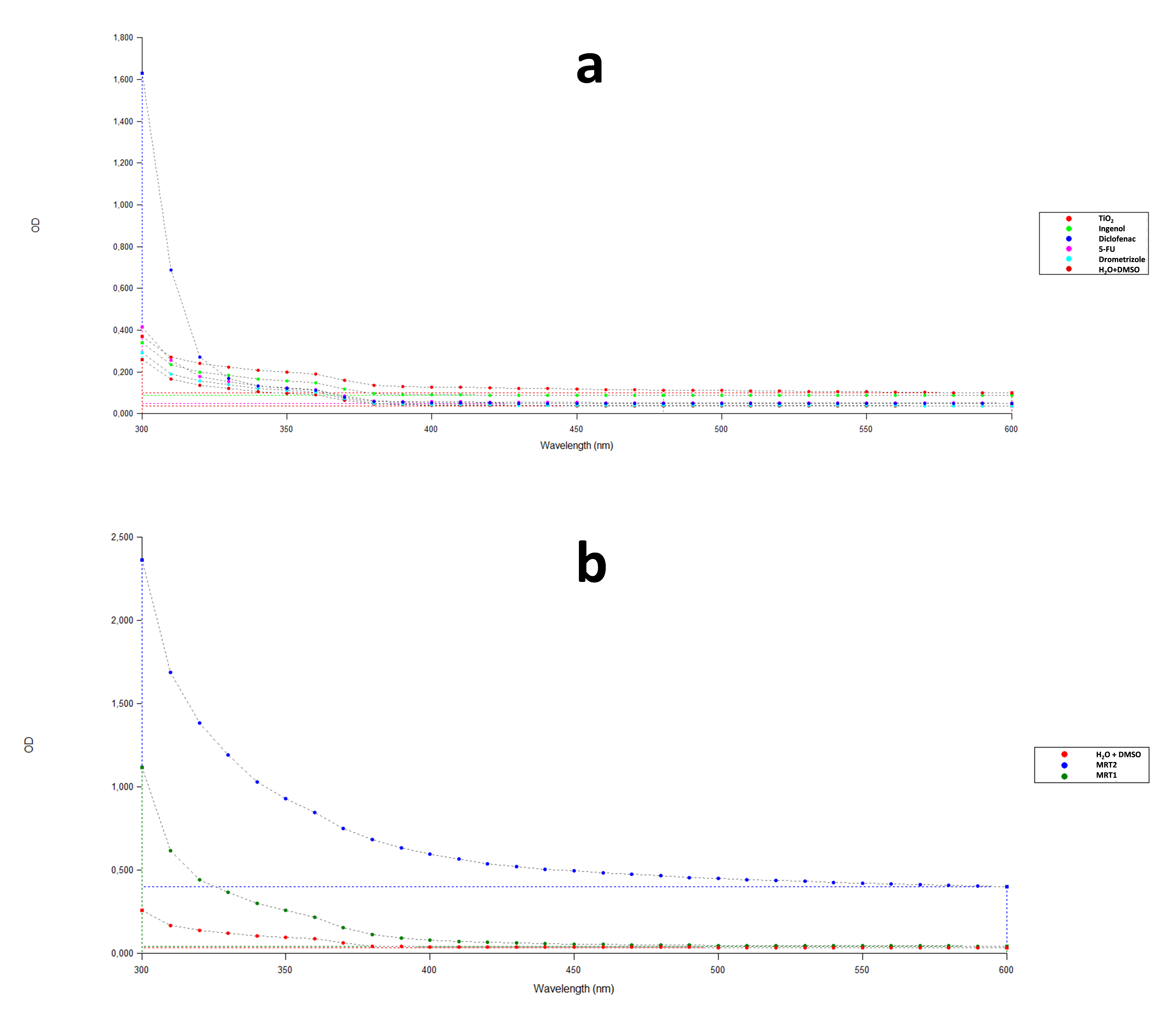

Supplement: S2 Fig — The peak of absorbance spectrum of used substances do not overlap with MTS/formazan peak absorbance between 450-500nm. (TIFF) [file pone.0184034.s002.tiff]

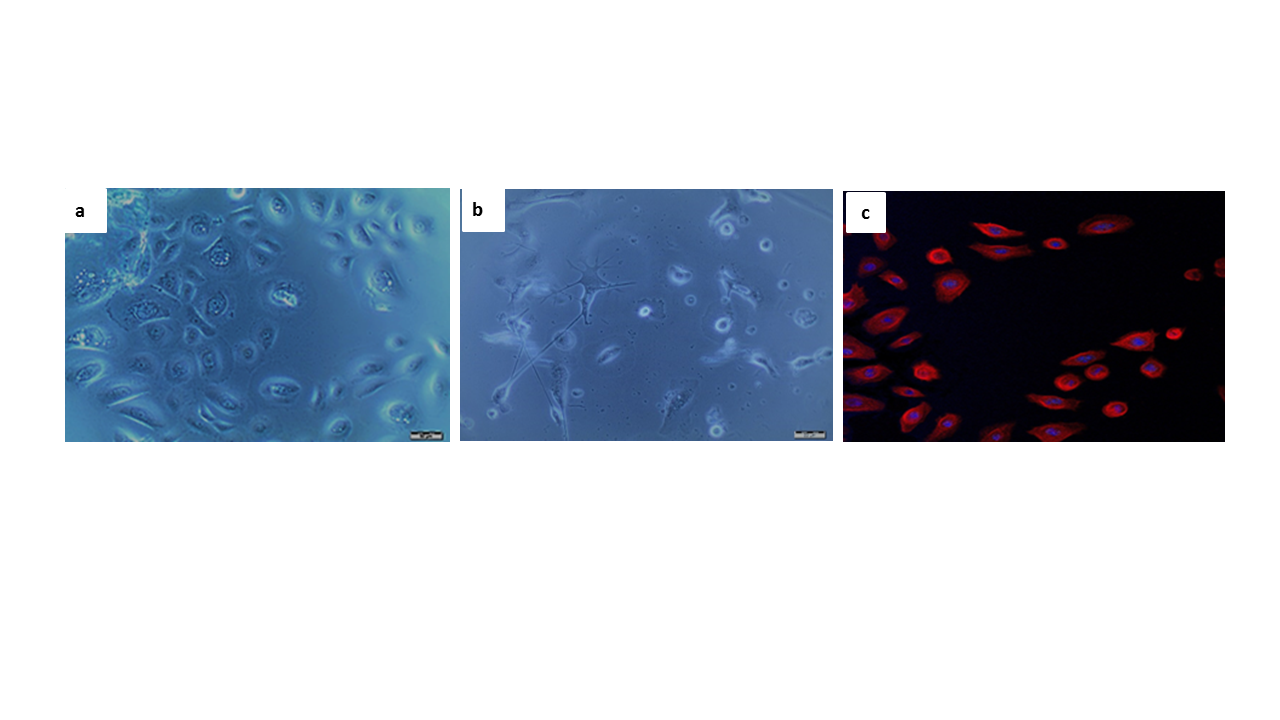

Supplement: S3 Fig — Magnification 100x. (TIF) [file pone.0184034.s003.tif]

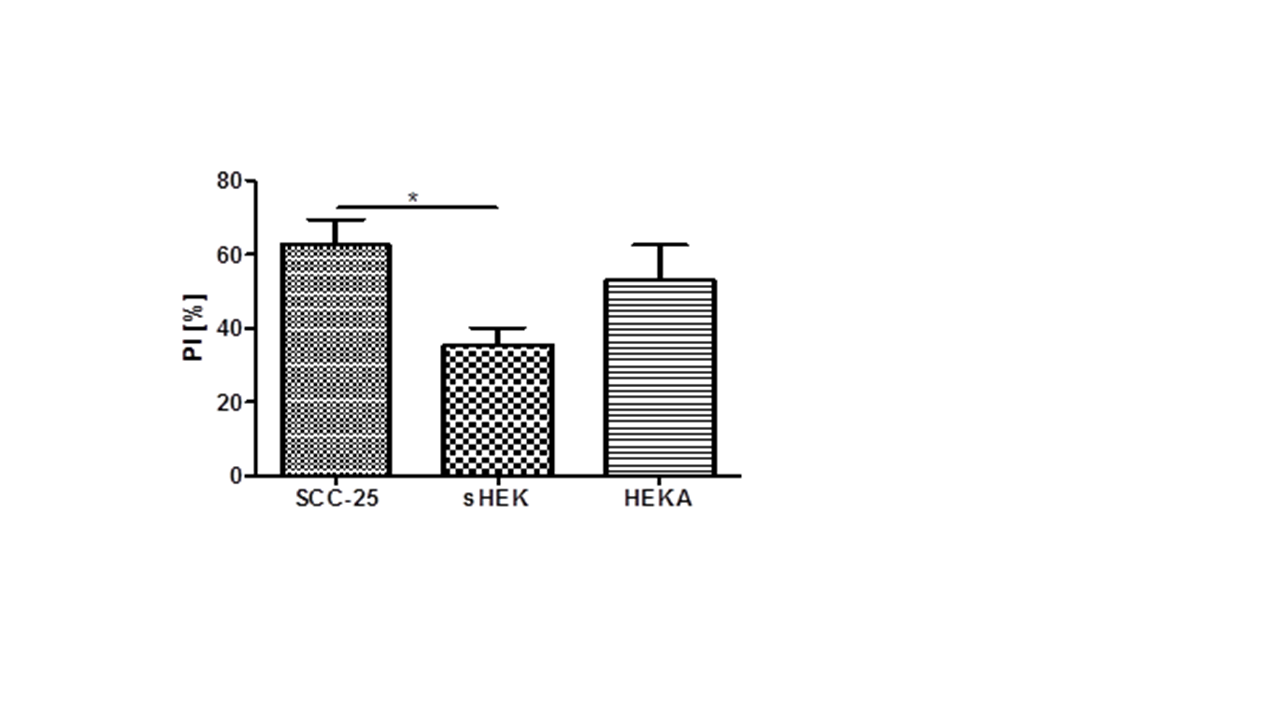

Supplement: S4 Fig — Statistical analysis used average values and comparison tests (ANOVA, Kruskal-Wallis/Dunn's Multiple Comparison tests) with Prism (* p<0,05). (TIF) [file pone.0184034.s004.tif]

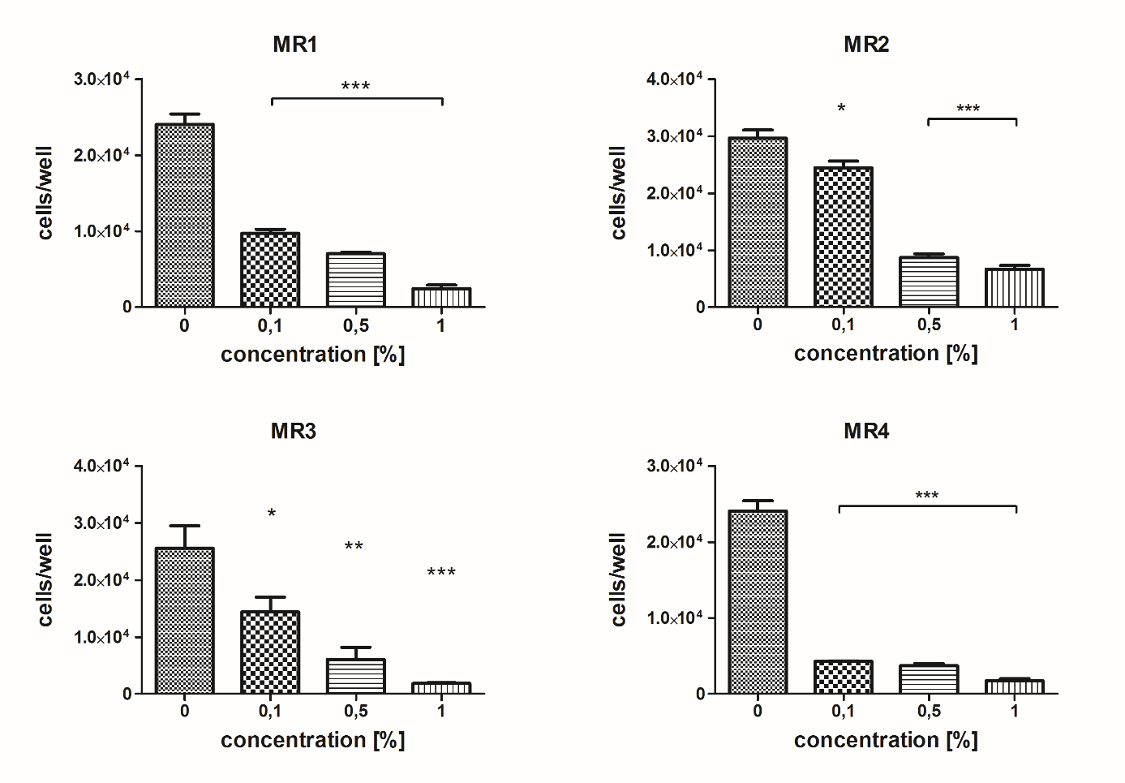

Supplement: S5 Fig — Statistical significance: (*) p<0.05, (**) p<0.01, (***) p<0.001. (TIFF) [file pone.0184034.s005.tiff]

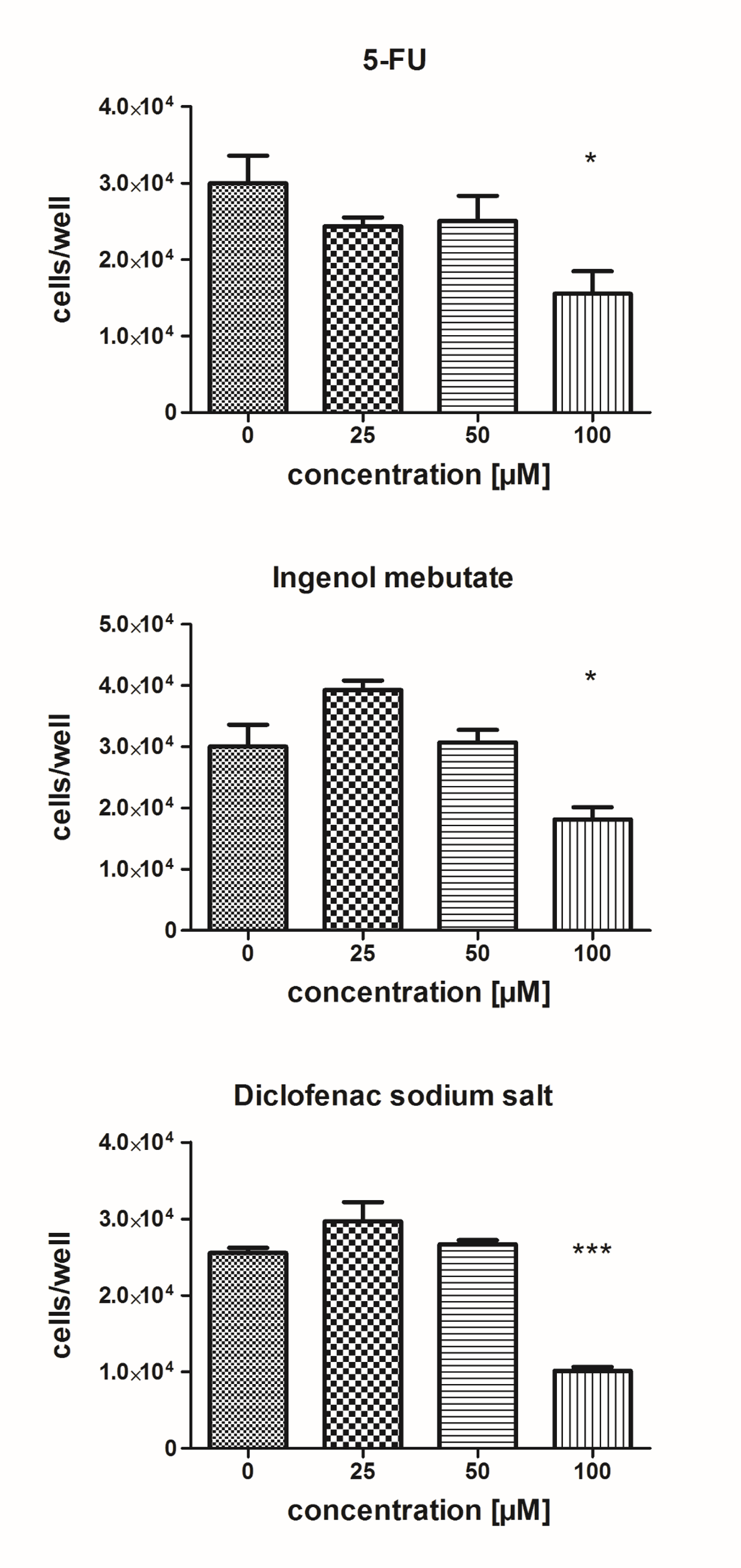

Supplement: S6 Fig — Statistical significance: (*) p<0.05, (**) p<0.01, (***) p<0.001. (TIFF) [file pone.0184034.s006.tiff]
