# Supplementary material for: The effect of wool hydrolysates on squamous cell carcinoma cells in vitro. Possible implications for cancer treatment
Source: PLoS One. 2017 Aug 31;12(8):e0184034. doi: 10.1371/journal.pone.0184034 (PMC5578621; doi:10.1371/journal.pone.0184034)
Supplement: S1 File — 0.85% NaCl served also as a blank. 1 mg of each sample was dissolved in 1 mL of 0.85% NaCl. 200 mL of each sample was transferred to 5 mL tubes followed by addition of 2.2 mL of Biuret reagent. Solution in each tube was stirred immediately and allowed to stand for 10 minutes. Next, 100 uL of Folin & Ciocalteu’s phenol reagent was added, obtained solution stirred and allowed to stand for 30 minutes. The solutions were subsequently transferred to 96-well plate and the absorbance was measured at a wavelength of 750 nm using Cytation3 microplate reader. Each of the sample was tested simultaneously in quadriplicate, and each of the experiments was repeated two times. (DOCX) [file pone.0184034.s007.docx]

To prepare the calibration curve, bovine serum albumin (BSA) in 0.85% NaCl was used as a standard. 0.85% NaCl served also as a blank. 1 mg of each sample was dissolved in 1 mL of 0.85% NaCl. 200 mL of each sample was transferred to 5 mL tubes followed by addition of 2.2 mL of Biuret reagent. Solution in each tube was stirred immediately and allowed to stand for 10 minutes. Next, 100 uL of Folin & Ciocalteu’s phenol reagent was added, obtained solution stirred and allowed to stand for 30 minutes. The solutions were subsequently transferred to 96-well plate and the absorbance was measured at a wavelength of 750 nm using Cytation3 microplate reader. Each of the sample was tested simultaneously in quadriplicate, and each of the experiments was repeated two times.
